# Feasibility of using administrative data to compare hospital performance in the EU

**DOI:** 10.1093/intqhc/mzu015

**Published:** 2014-02-18

**Authors:** O. Groene, S. Kristensen, O.A. Arah, C.A. Thompson, P. Bartels, R. Sunol, N. Klazinga, on behalf of the DUQuE Project Consortium, N Klazinga, DS Kringos, MJMH Lombarts, T Plochg, MA Lopez, M Secanell, R Sunol, P Vallejo, P Bartels, S Kristensen, P Michel, F Saillour-Glenisson, F Vlcek, M Car, S Jones, E Klaus, S Bottaro, P Garel, M Saluvan, C Bruneau, A Depaigne-Loth, C Shaw, A Hammer, O Ommen, H Pfaff, O Groene, D Botje, C Wagner, H Kutaj-Wasikowska, B Kutryba, A Escoval, A Lívio, M Eiras, M Franca, I Leite, F Almeman, H Kus, K Ozturk, R Mannion, OA Arah, M DerSarkissian, CA Thompson, A Wang, A Thompson

**Affiliations:** 1 Health Services Research, Faculty of Public Health and Policy, London School of Hygiene & Tropical Medicine, 15–17 Tavistock Place, London WC1H 9SH, UK; 2 Central Denmark Region & Center for Healthcare Improvements, Aalborg University, Denmark; 3 Department of Epidemiology, Fielding School of Public Health, University of California, Los Angeles (UCLA), Los Angeles, CA, USA; 4 UCLA Center for Health Policy Research, Los Angeles, California, USA; 5 Palo Alto Medical Foundation Research Institute, Palo Alto, CA, USA; 6 Central Denmark Region & The Department of Clinical Medicine, Aalborg University, , Denmark; 7 Avedis Donabedian Research Institute (FAD), Universitat Autonoma de Barcelona, Spain; 8 Red de investigación en servicios de salud en enfermedades crónicas REDISSEC, Spain

**Keywords:** audit, external quality assessment, quality management, quality indicators, measurement of quality, benchmarking, measurement of quality, health policy, health care system, safety indicators, patient safety, hospital care, setting of care

## Abstract

**Objective:**

To describe hospitals' organizational arrangements relevant to the abstraction of administrative data, to report on the completeness of administrative data collected and to assess associations between organizational arrangements and completeness of data submission.

**Design:**

A cross-sectional study design utilizing administrative data.

**Setting and Participants:**

Randomly selected hospitals from seven European countries (The Czech Republic, France, Germany, Poland, Portugal, Spain, and Turkey).

**Main Outcome Measures:**

Completeness of data submission for four quality indicators: mortality after acute myocardial infarction, stroke and hip fractures and complications after normal delivery.

**Results:**

In general, hospitals were able to produce data on the four indicators required for this research study. A substantial proportion had missing data on one or more data items. The proportion of hospitals that was able to produce more detailed indicators of relevance for quality monitoring and improvement was low and ranged from 40.1% for thrombolysis performed on patients with acute ischemic stroke to 63.8% for hip-fracture operations performed within 48 h after admission for patients aged 65 or older. National factors were strong predictors of data completeness on the studied indicators.

**Conclusions:**

At present, hospital administrative databases do not seem to be an appropriate source of information for comparison of hospital performance across the countries of the EU. However, given that this is a dynamic field, changes to administrative databases may make this possible in the near future. Such changes could be accelerated by an in-depth comparative analysis of the issues of using administrative data for comparisons of hospital performances in EU countries.

## Background

There is currently no source of information to compare the quality and safety performance of hospitals in the EU. Some countries in the EU produce comparative reports on hospital performance; however, the data often differ in terms of which hospitals participate, the type of service covered, indicator definitions, periodicity of reporting and feedback mechanism [1]. Even where similar indicators are reported, the actual data reported may have been subject to different risk-adjustments or may be reported in different formats [2].

The need for comparative data on hospital performance in EU countries is gaining importance as patients and purchasers contracting services seek providers delivering services of high quality, safety and efficiency. Directive 2011/24/EU of the European Parliament and The Council of the European Union (The Council) establishes the rights of EU patients to receive treatment in another EU country [3]. It stipulates that patients should have access to information on quality and safety of care to make an informed choice about their treatment and compare providers.

Administrative hospital data sources might be used to deliver such comparative analysis. In national contexts, it is increasingly being used to compare service utilization and outcomes, including mortality and complications, between hospitals [4]. Administrative data in health care is defined by ‘regular and continuing collection, use of standard definitions, some degree of obligation to collect the data completely and regularly [ … ] at national or regional level’ [5]. It is typically collected covering hospital inpatient activities for administrative purposes, in particular reimbursement. The clinical content of hospital administrative data includes only limited demographic characteristics and diagnoses of patients and codes for procedures [6]; however, since these data are readily available, are inexpensive to acquire, are computer readable and typically encompass large populations, they are often used to profile hospital activities, assess quality of care and investigate volume–outcome relationships. As administrative data are standardized at regional or national level and at its core are based on the International Classification of Diseases (ICD), they potentially allow a comparison of hospital performance between countries, even though these countries may not use the same ICD version [7].

The construction of quality indicators based on hospital administrative data was propelled by the Agency for Health Care Research and Quality (AHRQ) with the support of the University of California–Stanford Evidence-based Practice Center. In 1994, AHRQ released four sets of indicators, which were related to ambulatory care-sensitive conditions, inpatient quality, patient safety and pediatrics. These indicator sets, based on routinely collected hospital administrative discharge data and coded using the ICD, address a range of quality of care and patient safety concerns such as inpatient mortality for selected procedures and medical conditions, utilization of procedures for which there are concerns of overuse, underuse and misuse and volume of procedures addressing the volume–quality relationship, e.g. for esophagectomy or pancreatectomy [8].

The use of administrative data for quality assessment and improvement purposes has been criticized for its lack of specificity, varying coding practices that result in performance variations and lack of documentation on secondary diagnoses that impede risk adjustment. Problems with using administrative data for international benchmarking have been discussed previously, and data quality and unique personal identifiers are concluded to be a main issue. In addition, the task of retrieving, cleaning and presenting data is time-consuming and complex and requires skills and capacities at hospital level. However, revisions and improvements in accuracy of coding have substantially improved the validity of indicators derived from administrative data, even with the view to use them in international comparisons [9–12].

Given the potential future applications of administrative databases for comparisons of hospital performance, the objective of this paper was to describe hospitals' organizational arrangements relevant to the abstraction of administrative data, report on the completeness of administrative data collected in the eight countries and assess associations between the organizational arrangements and completeness of data submission.

## Method

### Setting and participants

The study took place in the context of the DUQuE project, which ran from 2010 to 2013 [13]. Respondents for this component of the DUQuE project were sampled from acute care hospitals in seven European countries (Czech Republic, France, Germany, Poland, Portugal, Spain and Turkey). In each country, 30 hospitals were randomly recruited if they had >130 beds and were treating patients with acute myocardial infarctions, hip fracture, stroke and mothers delivering babies. The conditions were chosen for their high financial volume, high prevalence of the condition, event rates (mortality and complications) and the different types of patients and specialists they were covering [14].

### Collection of administrative data

We collected data items to construct four outcome indicators: mortality after acute myocardial infarction, stroke, or hip fracture (for each, in-hospital mortality up to 30 days) and complications after vaginal delivery. Detailed data items requested from hospitals are presented in Table A1 in Appendix. Administrative data requests included: numbers of patients diagnosed with the condition (defined by ICD 9 and ICD 10 code, and by time period), numbers of patients who died (or suffered complication), age- and sex-specific stratifications of the denominator and details on the clinical management of relevance for quality improvement. In addition, we collected data at hospital level related to the context, use and coding of administrative data (see Table A2 in Appendix).

Indicators were derived from those used by the Organization for International Cooperation and Development (OECD). The OECD Health Care Quality Indicators Project is based upon data from national administrative data and comprises different sets of system-level indicator programs, e.g. the Regularly Collected and Times Series Indicators and Patient Safety Indicators [15] (Textbox 1). While a larger number of indicators were collected for the purpose of this study, we only report here on the key outcome measures for the four conditions: mortality after acute myocardial infarction, stroke and hip fractures and complications after normal delivery.

Textbox 1: **OECD Health Care Quality Indicators Project**

Since 2002 the Organization for International Cooperation and Development (OECD) has been developing, testing and reporting upon indicators that aim to compare quality of care between the member states. The program is coordinated through OECD's Health Care Quality Indicator committee that works at present with experts representing 33 countries. Results of the work have since 2005 bi-annually been published in Health at a Glance, lastly in November 2013 [16]. The present set of indicators focuses, among others, on potential preventable hospital admissions for chronic diseases (diabetes, chronic heart failure, COPD and asthma), 30-day case-fatality rates for patients admitted to a hospital for AMI or stroke, 48 h of operation time after hip fracture, survival-mortality and screening rates for breast, cervical and colon cancer, patient safety indicators (including indicators on obstetric trauma), excess mortality for patients with schizophrenia or bipolar disorders and a core set of patient experience questions. Many of the data in OECD's HCQI program are based on administrative data from hospitals, and a lot of development work has been done over the past years to understand and improve the comparability of these data between countries focusing on recording and coding quality as well as data-linkage capability and privacy concerns within member states [17–19].

All data collected pertained to the period of 1 January 2010 to 31 December 2010. A hospital coordinator retrieved feedback of electronic data from the administrative system and sent it to the project team for data cleaning and analysis. In most cases, hospitals were used for producing data abstracts as part of the national hospital administrative data collection. Abstraction of electronic data was done by a person with insight into the administrative data at hospital level, and according to standard procedures. This process was pilot-tested in two hospitals (one in Spain and one in Poland) before the initial data collection.

### Statistical analysis

We present descriptive statistics on characteristics of hospitals participating in the study, on hospital administrative functions related to data extraction and reporting and on data completeness for the four indicators used in this study. Data completeness is defined here in terms of the proportion of hospitals with complete data on the items required for the calculation of the indicator. We hypothesized that completeness of data submission was associated with various administrative functions and hospital characteristics, such as (i) whether data were abstracted systematically, (ii) whether the hospital database was part of a national database and (iii) whether the hospital database was used for reimbursement purposes, and (iv) hospital characteristics teaching status, ownership status or hospital size. To test our hypothesis, we used multivariate linear mixed regression with random intercept by country to account for clustering of hospitals within countries and adjusted for hospital size, ownership and teaching status and assessed the association between administrative functions and hospital characteristics and data completeness. All statistical analyses were carried out in SAS (version 9.3, SAS Institute Inc., NC, USA, 2011).

## Results

Overall, 177 hospitals contributed administrative data for the study (Table 1). About half (44.6%) of these were teaching hospitals and the majority (82.4%) under public ownership. Larger hospitals dominated the sample.

**Table 1:** Characteristics of hospitals participating in the analysis

Characteristic	*N* (%)
All hospitals	177 (100)
Czech Republic	28 (15.8)
France	25 (14.1)
Germany	13 (7.3)
Poland	30 (16.9)
Portugal	22 (12.4)
Spain	30 (16.9)
Turkey	29 (16.3)
Teaching hospitals	79 (44.6)
Public hospitals	146 (82.4)
Approximate number of beds in hospital	
<200	18 (10.1)
200–500	72 (40.6)
501–1000	60 (33.8)
>1000	27 (15.2)

We assessed generic issues regarding the organization and use of hospital administrative data (Table 2). Roughly two-thirds (63.8%) of hospitals kept separate records for patients seen at the ambulatory clinic and patients admitted to the hospital; however, this varied substantially between countries with country-level hospital averages ranging from 13.3 to 100%. The majority of hospitals (84.9%) had established a mechanism to systematically link patient-level information from outpatient and inpatient episodes of care. In most hospitals (84.7%), the administrative databases were used for reimbursement purposes.

**Table 2:** General questions (*N* = 177)

Survey question		All hospitals, *N* (%)	Country range (%)
A medical record is kept for every patient treated in the hospital	Yes	173 (97.7)	95.4–100
	No	2 (1.1)	
	Unknown	2 (1.1)	
Separate medical records are kept for patients seen at the ambulatory clinics at the hospital and patients admitted to the hospital	Yes	113 (63.8)	13.3–100
	No	55 (31.0)	
	Unknown	9 (5.0)	
If yes, there is a mechanism to systematically link or combine the information on the same patient acquired during ambulatory care visits (at the hospital) and during hospital admissions	Yes	96 (84.9)	75.0–92.8
	No	12 (10.6)	
	Unknown	5 (4.4)	
Data is systematically abstracted from the medical record into the hospital database	Yes	160 (90.3)	64.0–100
	No	6 (3.3)	
	Unknown	11 (6.2)	
The hospital database is part of a national database	Yes	82 (46.3)	7.6–86.3
	No	73 (41.2)	
	Unknown	22 (12.4)	
That hospital database is used for reimbursement purposes	Yes	150 (84.7)	55.1–100
	No	12 (6.7)	
	Unknown	15 (8.4)	
An ICD coding system is used in the hospital	Yes	169 (95.4)	82.1–100
	No	6 (3.3)	
	Unknown	2 (1.1)	

We report on the completeness of data submission for four indicators (AMI mortality, stroke mortality, hip fracture mortality and complications after delivery) (Table 3). The majority of hospitals contributed data for the calculation of the generic indicators. However, a substantial proportion had missing data on at least one of the data items required for the calculation of the indicator. This proportion ranged from 9.0% for the delivery complications indicator to 25.4% for the stroke mortality indicator. In general, most hospitals were able to provide data stratified by sex, and to a lesser extent, by age or were able to indicate length of stay.

**Table 3:** Analysis of completeness of data submission^a^

Data item contributions	AMI mortality *N* (%)	Stroke mortality, *N* (%)	Hip fracture, *N* (%)	Delivery complication, *N* (%)
Hospitals that contributed data for the calculation of the DUQuE indicators	168 (94.9)	171 (96.6)	172 (97.1)	168 (94.9)
Hospitals that contributed data but had missing data on any of the data items required for the calculation of the indicator	31 (17.5)	45 (25.4)	30 (16.9)	16 (9.0)
Hospitals that contributed, and were able to stratify by sex	161 (90.9)	155 (87.5)	167 (94.3)	N/A (N/A)
Hospitals that contributed and were able to stratify by age	148 (83.6)	137 (77.4)	159 (89.8)	143 (80.7)
Hospitals that contributed, and were able to indicate length of stay	153 (86.4)	146 (82.4)	158 (89.2)	N/A(N/A)

^a^Data completeness defined here as the proportion of hospitals with complete data on the items required for the calculation of the indicator.

In addition, we solicited information to calculate condition-specific indicators that are of particular relevance for quality assessment and improvement efforts. The proportion of hospitals that was able to provide this information was much lower than those for the provision of generic mortality and complications indicators: 53.1% (94) for percutaneous coronary interventions performed on patients diagnosed with myocardial infarction, 40.1% (71) for thrombolysis performed on patients with acute ischemic stroke, 63.8% (113) for hip fracture operations performed within 48 h after admission for patients aged 65 or older and 55.9% (99) for 3rd or 4th degree laceration where the child was delivered vaginally without help of any instruments (55.3% (98) in case with the use of instruments).

We assessed whether completeness of data submission was related to various administrative functions, such as (i) whether data were abstracted systematically, (ii) whether the hospital database was part of a national database and (iii) whether the hospital database was used for reimbursement purposes and (iv) hospital characteristics, such as teaching or ownership status or hospital size (Table 4). Hospital characteristics or systematic data abstraction were not associated with completeness of data submission in the statistical model. We did detect a significant and very strong positive association between the hospital database being part of a national database and the completeness of data submission for AMI completeness (*b* = 12.42, *P* = 0.02) and the hospital database being used for reimbursement purposes (*b* = 14.77, *P* = 0.069). However, we did not detect this positive association for the other indicators.

**Table 4:** Models for percent completeness as a function of administrative functions and hospital characteristics^a,b^

Predictor	AMI percent completeness			Stroke percent completeness			Hip percent completeness			Delivery percent completeness		
	*B*	se	*p*	*b*	se	*p*	*b*	se	*p*	*b*	se	*p*
Data is systematically abstracted from the medical record into the hospital database	1.27	11.38	0.911	8.43	11.32	0.457	0.13	9.69	0.989	11.36	11.92	0.342
The hospital database is part of a national database	12.42	5.30	0.020	−2.37	5.12	0.644	3.57	4.29	0.406	−5.34	5.43	0.327
The hospital database is used for reimbursement purposes	4.64	8.18	0.571	14.77	8.08	0.069	3.90	6.88	0.572	4.12	8.52	0.63
Public hospital	−7.82	7.21	0.280	2.26	6.67	0.735	−5.19	5.43	0.340	−3.86	7.15	0.589
Teaching hospital	−0.56	5.79	0.923	3.38	5.74	0.557	2.13	4.90	0.664	6.86	6.05	0.258
Hospital size												
<200	Ref	Ref	Ref	Ref	Ref	Ref	Ref	Ref	Ref	Ref	Ref	Ref
200–500	−5.87	7.85	0.456	13.86	10.03	0.169	−0.63	8.48	0.940	14.24	10.60	0.181
501–1000	−3.03	8.48	0.721	7.68	7.43	0.303	2.51	6.30	0.691	−1.24	7.85	0.874
>1000	−9.57	10.26	0.352	9.57	7.43	0.199	3.90	6.29	0.536	0.59	7.85	0.939

^a^Data completeness defined here as the proportion of hospitals with complete data on the items required for the calculation of the indicator.

^b^Multivariate linear mixed model with random intercept by country; all adjustment variables displayed.

## Discussion

We reported on organizational arrangements for the abstraction of administrative data, completeness of administrative data and associations between organizational arrangements and completeness of data submission. The study revealed substantial differences in the national contexts of using hospital administrative data (e.g. whether the hospital database is part of a national database and whether this is used for the reimbursement of hospital services). We also identified major differences in hospital administrative data functions regarding the linkage of in- and outpatient data.

Hospitals in general were able to produce the data on four indicators required for this research study. However, a substantial proportion of hospitals had missing data on any of the data items, and not all hospitals were able to stratify on standard variables, such as age and sex, and ability to do so varied across indicators. More importantly, the proportion of hospitals that was able to produce more detailed indicators of relevance for quality monitoring and improvement was much lower. In a multivariate analysis, national factors such as the hospital database being part of a national database and the hospital database being used for reimbursement purposes were strong predictors for data completeness on selected indicators. However, this effect was not systematic across indicators.

This study has a number of limitations. First, although hospitals were sampled randomly, the study was not designed to generalize findings at country level. Second, while our main objective was to investigate issues related to data availability, we recognize that many issues need further investigation, especially coding quality (e.g. number of diagnostic and procedural information reported, availability of presence on admission indicators, and distinguishing transfers from another acute care facility to the reporting hospital). Third, we intentionally focused on fairly crude data and did not distinguish between country requirements to report certain data items. Finally, we were not able to assess the capabilities of data custodians to facilitate data extraction. The last two issues would be of particular importance if had we aimed to establish findings that are generalizable for each of the participating countries. In contrast, we were interested to know whether individual hospital had the capacity to provide data to satisfy the definitions for the commonly used quality and safety indicators pursued here.

These results have important implications regarding the potential future use of hospital administrative databases for pan-European comparisons of hospital performance. At current, it seems that hospital comparisons would be feasible using generic indicators only. Some national health systems context appear to be more supportive of such comparisons as data completeness appears better; however, the proportion of hospitals able to produce specific indicators overall was very low.

Administrative databases so far have mostly been used within regional/national boundaries, and initiatives to compare hospital performance internationally are limited. Recent advances to include ‘present on admission’ indicators in the USA or in Belgium allow to compute indicators that address important quality and safety concerns, such as pressure sores acquired during hospital stay [20]. In addition, hospital performance data can be linked to national mortality databases to provide information on long-term outcomes and survival, provided data can be tracked across providers, which is greatly facilitated by unique person identifiers [21]. Given these advances, performance indicators based on administrative data are a very useful tool to flag potential quality problems in hospitals. Yet, our analysis shows that simple data quality issues are a major hurdle for the feasibility of such comparisons.

Other initiatives aim to facilitate international comparisons of hospital performance; however, their coverage is very biased (as most participants tend to be those already performing well) and limit participation (due to the cost implications of participating) [22–24]. Therefore, in the medium- to long-term, it is likely that hospital administrative data could play an important role to provide comparative information on hospitals performance for patients seeking care and providers contracting services. Particularly given the release of ICD 11 in 2015, which includes an enhanced classification to capture health care quality and patient safety issues, including ‘(i) causes of harm (medications, procedures, devices or other aspects of care), (ii) mode or mechanism of harm linked to each of the coded causes of harm and (iii) the harm incurred (coded from any of the codes available throughout the classification)' [25].

Before comparisons of hospital performance are possible, however, more research on hospital administrative data should address the following issues within the national context of (EU) countries: coverage of the administrative database (geographical and hospital type), levels of case ascertainment, scope of variables included in the database, completeness of data, use of unique personal identifiers, reliability of coding of conditions and interventions, explicit definitions for variables, use of explicit rules for deciding how variables are recorded, coding of secondary diagnosis, independence of observation of primary outcome and extent to which data are validated [26, 27]. Such an overview would help establishing a roadmap toward using administrative data to compare hospital performance in EU countries.

## Conclusion

Hospitals participating in the study were able to extract and submit data from their administrative systems to produce basic quality indicators. The completeness of data submitted was low, especially for specific quality indicators, and hospital's ability to perform simply stratifications of data was limited. At current, hospital administrative databases do not seem to be an appropriate source of information for comparisons of hospitals performance in the EU. However, given that this is a dynamic field, it might be possible in the medium- to long-term. In the meantime, research should target an in-depth comparative analysis of the issues of using administrative data for comparisons of hospital performances in EU countries.

## Funding

The study, “Deepening our Understanding of Quality Improvement in Europe (DUQuE)” has received funding from the European Community's Seventh Framework Programme (FP7/2007–2013) under grant agreement n° 241822. Funding to pay the Open Access publication charges for this article was provided by the European Commission under grant agreement 241822.

## Appendix

[Table MZU015TBA1]

**Table A1 MZU015TBA1:** Condition-specific administrative data items requested from participating hospitals

**Acute myocardial infarction**
1. Number of cases discharged by diagnosis (ICD 9 410 or ICD 10 I21 and I22)
2. Number of cases by diagnosis (as above) and age groups
3. Number of cases by diagnosis (as above) and sex
4. In hospital mortality (up to 30 days)
5. Length of stay (specified as min, max and median)
6. Number of percutaneous coronary intervention performed
**Stroke**
1. Number of acute cases discharged by diagnosis (ischemic stroke and not specified stroke: ICD 9 433.01, 433.11, 433.21, 433.31, 433.91, 434.01, 434.11 and 433.91or ICD 10 I63 and I64) and (hemorrhagic stroke ICD 9 431 or ICD 10 I61)
2. Number of cases by diagnosis (as above) and age groups
3. Number of cases by diagnosis (as above) and sex
4. In hospital mortality per diagnosis (up to 30 days)
5. Length of stay (min, max and median) per diagnosis (as above)
6. Number of patients discharged from specialized stroke units, medical wards, neurological wards, rehabilitation wards, non-specific medical ward, ICU and other
7. Number of thrombolysis performed
**Hip fracture**
1. Number of acute cases discharged by diagnosis (ICD 9 8200-1 or ICD S72.0) and (ICD 9 8202-3 or ICD 10 S72.2)
2. Number of cases by diagnosis (as above) and age groups
3. Number of cases by diagnosis (as above) and sex
4. In hospital mortality per diagnosis (up to 30 days)
5. Length of stay (min, max and median)
6. Number of hip-fracture operations performed
**Deliveries**
1. Number of deliveries
2. Number of cases by age groups
3. In hospital newborn mortality (up to 30 days)
4. Length of stay (min, max and median) for the mother
5. Length of stay (min, max and median) for the child
6. Number of singleton
7. Number of births with two or more children
8. Number of newborns transferred to neonatal care/ICU
9. Number of cesarean sections

[Table MZU015TBA2]

**Table A2 MZU015TBA2:** General information related to the use of administrative data requested from participating hospitals

Question	Response category
Do you keep a medical record for every patient treated in your hospital?	0. No
	1. Yes
	9. Don't know
Do you keep separate medical records for patients seen at the ambulatory clinics at the hospital and patients admitted to the hospital?	0. No
	1. Yes
	9. Don't know
If yes, is there a mechanism to systematically link or combine the information on the same patient acquired during ambulatory care visits (at the hospital) and during hospital admissions?	0. No
	1. Yes
	9. Don't know
Do you systematically abstract data from the medical record into a hospital database?	0. No
	1. Yes
	9. Don't know
Is that hospital database part of a national database?	0. No
	1. Yes
	9. Don't know
Is that hospital database used for reimbursement purposes?	0. No
	1. Yes
	9. Don't know
Do you use an ICD coding system in your hospital?	0. No
	1. Yes
	9. Don't know
ICD diagnosis codes used for acute myocardial infarction	ICD codes for acute myocardial infarction
ICD diagnosis codes used for stroke (ischemic and hemorrhagic stroke)	ICD codes for stroke
ICD diagnosis codes used for hip fractures	ICD codes for hip fractures
